# Reduced mitochondrial size in hippocampus and psychiatric behavioral changes in the mutant mice with homologous mutation of *Timm8a1-I23fs49X*

**DOI:** 10.3389/fncel.2022.972964

**Published:** 2022-08-25

**Authors:** Niemtiah Ouattara, Zirui Chen, Yihua Huang, Xia Chen, Pingping Song, Zhongju Xiao, Qi Li, Yuqing Guan, Ziang Li, Yawei Jiang, Kaibiao Xu, Suyue Pan, Yafang Hu

**Affiliations:** ^1^Department of Neurology, Nanfang Hospital, Southern Medical University, Guangzhou, China; ^2^Department of Clinical Laboratory, Nanhai District People’s Hospital of Foshan, Foshan, China; ^3^Department of Neurology, First Affiliated Hospital, Jinan University, Guangzhou, China; ^4^Department of Physiology, School of Basic Medical Sciences, Southern Medical University, Guangzhou, China; ^5^Department of Otolaryngology-Head and Neck Surgery, Nanfang Hospital, Southern Medical University, Guangzhou, China

**Keywords:** deafness-dystonia-optic neuronopathy (DDON) syndrome, DDP1, *TIMM8A*, MTFP1, mitochondrial fission

## Abstract

**Background:**

Deafness-dystonia-optic neuronopathy (DDON) syndrome, a condition that predominantly affects males, is caused by mutations in *translocase of mitochondrial inner membrane 8A* (*TIMM8A*)*/deafness dystonia protein 1* (*DDP1*) gene and characterized by progressive deafness coupled with other neurological abnormalities. In a previous study, we demonstrated the phenotype of male mice carrying the hemizygous mutation of *Timm8a1-I23fs49X*. In a follow-up to that study, this study aimed to observe the behavioral changes in the female mutant (MUT) mice with homologous mutation of *Timm8a1* and to elucidate the underlying mechanism for the behavioral changes.

**Materials and methods:**

Histological analysis, transmission electron microscopy (EM), Western blotting, hearing measurement by auditory brainstem response (ABR), and behavioral observation were compared between the MUT mice and wild-type (WT) littermates.

**Results:**

The weight of the female MUT mice was less than that of the WT mice. Among MUT mice, both male and female mice showed hearing impairment, anxiety-like behavior by the elevated plus maze test, and cognitive deficit by the Morris water maze test. Furthermore, the female MUT mice exhibited coordination problems in the balance beam test. Although the general neuronal loss was not found in the hippocampus of the MUT genotype, EM assessment indicated that the mitochondrial size showing as aspect ratio and form factor in the hippocampus of the MUT strain was significantly reduced compared to that in the WT genotype. More importantly, this phenomenon was correlated with the upregulation of translation of mitochondrial fission process protein 1(Mtfp1)/mitochondrial 18 kDa protein (Mtp18), a key fission factor that is a positive regulator of mitochondrial fission and mitochondrial size. Interestingly, significant reductions in the size of the uterus and ovaries were noted in the female MUT mice, which contributed to significantly lower fertility in the MUT mice.

**Conclusion:**

Together, a homologous mutation in the *Timm8a1* gene caused the hearing impairment and psychiatric behavioral changes in the MUT mice; the latter phenotype might be related to a reduction in mitochondrial size regulated by MTP18.

## Introduction

Deafness-dystonia-optic neuronopathy (DDON) syndrome [Mendelian Inheritance in Man (MIM) 304700], also named Mohr-Tranebjaerg syndrome (MTS), is a rare X-linked recessive neurodegenerative disorder characterized by progressive deafness in early childhood followed by increased dystonia, ataxia, and visual deficit in adulthood or adolescence (Tranebjaerg et al., 1995). Other psychiatric abnormalities frequently reported to be associated with DDON syndrome include dementia occurring around 40 years of age, irritability, and mental disturbance. The causative gene is *translocase of mitochondrial inner membrane 8A* (*TIMM8A*)*/deafness dystonia protein 1* (*DDP1*), which is located on Xq22.1 (Tranebjærg, 1993; Tranebjaerg et al., 1995; Jin et al., 1996). Most of the disease-related mutations are frameshift or nonsense mutations leading to premature stops, while there are a few missense mutations (Penamora-Destriza et al., 2015; Wang et al., 2019). Deafness-dystonia-optic neuronopathy syndrome mostly affects males and is less common among females due to X chromosome inactivation. However, there are a few female carriers who have developed symptoms later in life and present with mild hearing impairment and dystonia (Swerdlow and Wooten, 2001; Ha et al., 2012; Tranebjærg, 2013).

Although DDON syndrome is thought to be caused by a defect in the mitochondrial import machinery, pathomechanism has not yet been elucidated. Tim8 belongs to the conserved small zinc-finger protein family, consisting of Tim8, 9, 10, 12, and 13 in yeast and hTim8a, 8b, 9, 10, 12, and 13 in humans. The small Tim family resides in the intermembrane space (IMS) of mitochondria. In yeast, the Tim9-10-12 complex is essential, but the Tim8–13 hexameric complex is only required to import the translocase of the inner membrane (TIM) protein Tim23 under compromised conditions (Paschen et al., 2000). Importing of the translocase of the outer membrane (TOM) protein Tom40 by the Tim8–13 complex has been also revealed in yeast (Hoppins and Nargang, 2004; Wiedemann et al., 2004; Habib et al., 2005). In agreement with previous studies on yeast, the transfected human missense mutant DDP1-C66W in yeast incorrectly folded and disrupted the assembly of Tim8–13 (Hofmann et al., 2002). In isolated mouse liver mitochondria, hTim8a–13 facilitates the import of hTim23 (Rothbauer et al., 2001). In addition, in lymphoblast cell lines from a patient with DDP1-Q34X, the import of the matrix-targeted Su9-DHFR, Tim23, and citrin fragment, and NADH levels were decreased, indicating a general defect in import and metabolic impairment (Roesch et al., 2004). However, we and others did not find Tim8a to mediate the biogenesis of Tim23, nor Tom proteins. Lack of DDP1 in the mitochondria of the fibroblasts isolated from patients with DDON syndrome harboring DDP1-Q38fs64X did not affect the expression levels of hTom40 or hTim23 (Engl et al., 2012). The Tom40 level was stable in the neuronal mitochondria from *Timm8a1*^*I*23*fs*49*X/Y*^ mutant mice (Song et al., 2020). In neuroblastoma cell SH-SY5Y, loss of hTim8a led to assembly defects of complex IV, which primer SH-SY5Y cells to apoptosis (Kang et al., 2019). Thus, further study will provide clues for understanding the function of Tim8a in the mitochondria.

Since most patients with DDON syndrome are males, our previous study has reported that the male *Timm8a1*^*I*23*fs*49*X/Y*^ mutant mice exhibited less weight gain, hearing impairment, and cognitive deficit (Song et al., 2020). This follow-up study aimed to analyze the behavioral changes in the female mice with homologous mutation (MUT) in the *Timm8a1* gene. We found that the female MUT mice similarly showed hearing impairment and cognitive impairment and anxiety-like behavior. The latter two phenotypes in the MUT mice might be correlated with a reduced mitochondrial size and an upregulated level of mitochondrial fission process protein 1(Mtfp1)/mitochondrial 18 kDa protein (Mtp18), which is a positive regulator of mitochondrial fission and mitochondrial size (Tondera et al., 2005).

## Materials and methods

### Animals

All animal experiments have been approved by the Animal Ethics Committee of Southern Medical University, China, and were conducted following the guidelines for the care and use of laboratory animals. Generation of female mice with a homologous mutation in the *Timm8a1* gene was done by breeding the *Timm8a1*^*I*23*fs*49*X/Y*^ hemizygous MUT mice (Song et al., 2020) with female mice carrying the heterozygous Tim8a1 mutation. All *Timm8a1* mutant mice and wild-type (WT) littermates were bred under the standard environment with the temperature at 22 ± 1°C, the humidity around 55–70%, the cycle of light and darkness in 12 h, and the stocking density at ≤5 animals/cage. Mice had access to food and water *ad libitum*.

### Behavioral studies

All behavioral tests were performed and analyzed by two technicians who were blinded with the mouse genotype. The female MUT mice, *Timm8a1*^*I*23*fs*49*X*/*I*23*fs*49*X*^, and the female WT mice were evaluated. Before any test, all animals were familiarized with the testing room for 30 min to fit into the environment. According to the previous report (Song et al., 2020), all animal behavioral tests were carried out during the daytime.

### Auditory brainstem response test

The auditory brainstem response (ABR) test is a widely used method to measure the electrical responses of auditory activity in humans or rodents. Since the auditory function of mice matures earlier, we took 4–5 week-old juvenile mice for ABR testing. As previously reported, the mice were stimulated with tone pips or clicks using a Tucker–Davis Technologies workstation System III, which was installed using the SigGen32 software (TDT, Alachua, FL, United States), respectively. Both stimuli ranged from 10 to 80 dB at 5 dB intervals. The duration of the click stimuli was 4 ms with 0.5 ms rising and falling times. The click frequencies were 8, 16, and 32 kHz. The stimuli duration was 0.1 ms. The thresholds were recorded.

### Balance beam test

Balance and motor coordination were measured on the balance beam at 9–10 weeks. The beam apparatus was 0.9 cm wide, 100 cm long, and 60 cm high. It was installed between two tables, with a black box (20 cm × 20 cm) placed at the end of the beam. The black box was filled with toys on the training days and was empty on the probe test day. The training took three consecutive days. On the fourth day, the probe test was performed three times for each mouse, and a video camera was used to record the time for each mouse to cross the beam and the times of rear paws slips off the beam.

### Accelerating rotarod test

The rotarod test was assessed on the rotarod equipment (TSE, Homburg, Germany). Each mouse was trained for three consecutive days and tested on the fourth day. The accelerated rate ranged from 4 to 40 rpm within 5 min. The rod time was recorded automatically for each mouse, and the average time was counted.

### Elevated plus maze test

The elevated plus maze test is another assessment to evaluate anxiety-like behavior. The elevated plus maze apparatus consisted of two open arms (30 cm × 5 cm), two closed arms with no roof (30 cm × 5 cm × 20 cm), and an open square (5 cm × 5 cm) in the center. The maze was 50 cm above the floor. Mice were put in the central area with their head toward the open arm. The times of each mouse entering the open arms and the duration of each mouse staying in open arms were recorded in a 5-min test trial.

### Morris water maze test

The Morris Water Maze test was used to measure spatial-dependent learning and memory. The equipment (TSE Co., Thuringia, Germany^1^) consisted of a circular pool (110 cm in diameter and 60 cm in depth) filled with milk water and a floating hidden platform (8 cm in diameter, 30 cm in height, and 1 cm below the water surface). The swimming path was recorded using the TSE video system. During the 4-day training stage, each mouse was placed in the water for 60 s to escape onto the platform. If the mouse was once unable to find the platform within 60 s, then it was removed and put on the platform for 15 s to remember the location. On the 5th day (probe test), the platform was removed, and each mouse was put in the pool for a free swim for 60 s. The latency spending in the target quadrant and the times crossing the platform position were recorded. The mice in the two groups were tested alternatively to minimize the impact of the day’s different times.

### Histological staining and immunohistochemistry

The organs of the mice were obtained and fixed in 4% paraformaldehyde and kept at 4°C for 24 h, and then transferred to ethyl alcohol for gradient dehydration before being embedded in paraffin. The mouse brains were sliced (4 μm per section) using the Leica RM2245 vibratome (Leica Microsystems, Heidelberg, Germany) and sent for further immunohistochemistry. Sections were incubated with anti-NeuN rabbit monoclonal antibody (Ab, Abcam, ab177487, Waltham, MA, United States) at a 1:1,000 dilution. After washing with saline, goat anti-rabbit secondary Ab conjugated with horseradish peroxidase (HRP) (ZSGB-Bio, Beijing, China) was applied and further visualized with diaminobenzidine (DAB) substrate solution (ZSGB-Bio, Beijing, China) under a microscope (LEICA DM 3000 LED, Germany). The ovaries were serially sliced (5 μm per section), dewaxed, and rehydrated in xylene and ethyl alcohol, followed by H&E staining. All pictures were taken under an optical microscope.

### Western blotting analysis

Each mouse was sacrificed after anesthesia, and the hippocampus was homogenized in Radio immunoprecipitation assay buffer, Radio immunoprecipitation assay (RIPA) lysis buffer reagent (Beyotime Biotechnology, Shanghai, China) using a homogenizer. Notably, 30 μg of total proteins were electrophoresed through 12% Bis-Tris sodium dodecyl sulfate (SDS, CH3(CH2)11OSO3Na) polyacrylamide gels and then transferred to a polyvinyl difluoride (PVDF) membrane. The primary Abs were anti-MTP18 Ab (Proteintech, 14257-1-AP, Rosemont, IL, United States), anti-DRP1 Ab (Abcam, ab184247), and anti-beta-actin Ab (Bioss, bs-0061R, Boston, MA, United States). Goat anti-rabbit IgG (H + L)-HRP (Bioworld, BS13278, Bloomington, IN, United States) was used as the secondary Ab and protein signals were detected using the enhanced chemiluminescence (ECL) system (CWBIO, Taizhou, Jiangsu, China).

### Transmission electron microscopy analysis

The ultrastructure of mitochondria in the hippocampus of the mice at 6 months old was observed under electron microscopy (EM). Each mouse was sacrificed after anesthesia, and the hippocampus was fixed in 2.5% glutaraldehyde, embedded in Epon 812, and cut into 70-nm thick slices, then stained with uranyl acetate and lead citrate. Finally, the images of mitochondrial ultrastructure were captured using a Hitachi H-7,650 EM (Hitachi, Tokyo, Japan). Using National Institutes of Health (NIH) ImageJ, 10 fields of view (magnification of 30,000×) were randomly selected to assess the aspect ratio (ratio of major and minor axis) of individual mitochondria. The perimeter and area of the mitochondria were measured from five fields. The form factor representing the degree of mitochondrial branching is defined as [(perimeter^2^)/(4π × area)] (Abuarab et al., 2017).

## Statistical analysis

All data are presented as means ± SD. The result of average litter size was analyzed using the Mann–Whitney test, and other data were carried out using the *t*-test. Statistical analysis was conducted using the SPSS software version 21 (SPSS, Chicago, IL, United States) and GraphPad Prism 8 (Graph Pad, La Jolla, CA, United States). A *P-*value of <0.05 was considered to be statistically significant.

## Results

### Less weight gain in the female mutant mice

**FIGURE 1:**
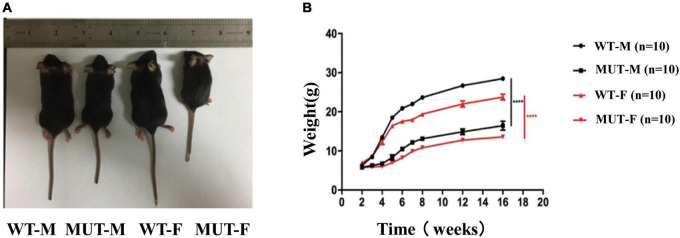
The female mice carrying the homologous mutation of *Timm8a1-I23fs49X* exhibited less gain of weight. **(A)** Mice at the age of 4–5 weeks [from left to right: wild-type (WT) male, MUT male, WT female, MUT female mouse]. **(B)** Weekly bodyweight. The *Timm8a1* MUT mice were significantly smaller than the WT mice since the 3rd week (^****^p < 0.0001).

Previously, we have observed that the male *Timm8a1*^*I*23*fs*49*X/Y*^ mutant mice presented with less weight gain (Song et al., 2020). Here, we compared the body size of the WT mice and MUT mice for 4–5 weeks, suggesting that the latter are much smaller than the former (Figure 1A). Figure 1B shows that both male and female MUT mice had less gain of weight following the age of 3 weeks. Meanwhile, the MUT mice survived similarly to the WT mice.

### The female *Timm8a1*^*I*23*fs*49*X*/*I*23*fs*49*X*^ mutant mice had hearing impairment

**FIGURE 2:**
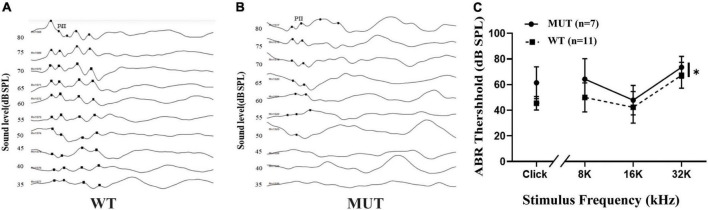
*Timm8a1*^*I*23*fs*49*X/I*23*fs*49*X*^ MUT mice had hearing impairment at an early age. **(A)** Representative auditory brainstem responses (ABRs) (to 16 kHz pure-tone stimuli) of wild-type (WT) mice at the age of 4–5 weeks. Five positive peaks of the waveforms are shown, and wave two was labeled as PII. **(B)** Representative ABRs (to 16 kHz pure-tone stimuli) of MUT mice at the age of 4–5 weeks. **(C)** Thresholds for ABRs evoked by clicks and 8–32 kHz pure-tone pips are shown for the WT mice and the MUT mice, suggesting that the MUT mice had elevated threshold of ABRs (WT, *n* = 11; MUT, *n* = 7, *p < 0.05). Error bars represent mean ± SD.

The ABR test provides information about the cochlea and the central pathways for hearing. We compared the hearing threshold between the WT and MUT mice at 4–5 weeks of age. The ABR test comprised click and several pure tone stimuli, including frequencies of 8, 16, and 32 kHz. The ABR thresholds that responded to the pure-tone stimuli at 16 kHz were compared between the WT mice (Figure 2A) and the MUT mice (Figure 2B). The WT mice showed a clear response starting from a sound level of 35 dB sound pressure level (SPL), whereas the MUT mice had a response at a sound level of 55 dB SPL. Compared with the WT mice, the *Timm8a1*^*I*23*fs*49*X/I*23*fs*49*X*^ MUT mice had significantly increased ABR thresholds stimulated by clicks at 8, 16, and 32 kHz (Figure 2C). Thus, homologous mutation of the *Timm8a1* gene leads to impaired hearing in female MUT mice at an early age.

### *Timm8a1*^*I*23*fs*49*X/I*23*fs*49*X*^ mice had balance problems and impaired maximal motor performance

**FIGURE 3:**
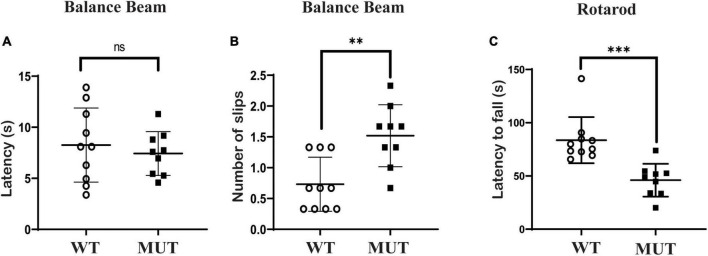
*Timm8a1*^*I*23*fs*49*X/I*23*fs*49*X*^ MUT mice presented with mildly impaired coordinative ability and impaired maximal motor performance. **(A,B)** Balance beam test. The MUT mice took a similar time to cross the beam compared to the wild-type (WT) mice (WT = 8.26 ± 3.63, *n* = 10, MUT = 7.43 ± 2.15, *n* = 9, *P* > 0.05), while the MUT mice slipped more frequently on the balance beam than the WT mice (WT = 0.73 ± 0.44, *n* = 10; MUT = 1.52 ± 0.50, *n* = 9, ^**^P < 0.01). **(C)** Rotarod test. The MUT mice stayed less time on the rod than the WT mice (WT = 83.65 ± 21.69, *n* = 10; MUT = 46.02 ± 15.44, *n* = 9, ^***^P < 0.001). Error bars represent mean ± SD.

Mice at the age of 9–10 weeks were tested on the balance beam and rotarod. On the balance beam test, the *Timm8a1*^*I*23*fs*49*X/I*23*fs*49*X*^ mice took the same time to cross the balance beam but slipped more times compared to the WT mice (Figures 3A,B). In addition, there was a significant difference in the rotarod test between the MUT mice and the WT mice (Figure 3C). Thus, the results indicate that female MUT mice had some degree of impaired coordination and motor performance.

### The female mutant mice had a mild memory deficit and increased anxiety-like behavior at the age of 20 weeks

**FIGURE 4:**
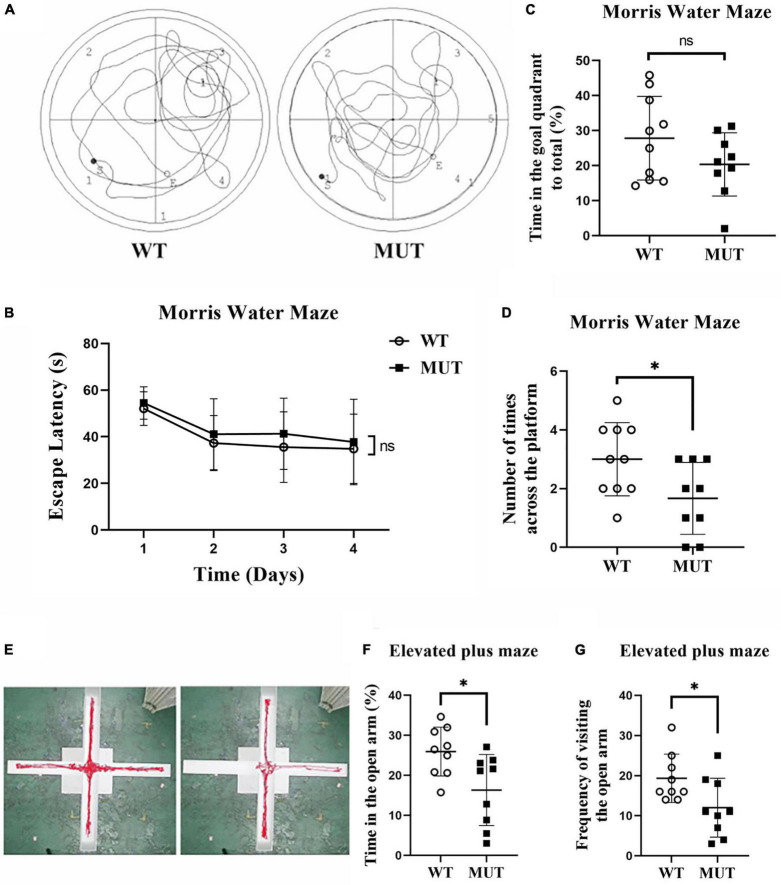
*Timm8a1*^*I*23*fs*49*X/I*23*fs*49*X*^ MUT mice had impaired learning and anxiety-like behavior at 20 weeks of age. **(A)** Traveling trace in the Morris Water Maze test. **(B)** From day 1 to day 4 of training, all mice showed a gradual decline in the latency to find the hidden platform over time. **(C)** On the probe test day, there was no difference in the spent time in the goal quadrant between the two groups (WT = 27.83 ± 11.95, *n* = 10; MUT = 20.34 ± 9.04, *n* = 9, *p* > 0.05)**. (D)** On the probe test day, the MUT mice crossed less frequently in the platform area than the wild-type (WT) mice (WT = 3.00 ± 1.25, *n* = 10; MUT = 1.67 ± 1.22, *n* = 9, *p < 0.05). **(E)** Trace of the WT and MUT mice in the elevated plus maze test for 5 min. **(F)** Elevated plus maze test. The MUT mice spent less time in the open arm than the WT mice (WT = 25.93 ± 6.11, *n* = 9; MUT = 16.32 ± 8.87, *n* = 9, *p < 0.05). **(G)** Elevated plus maze test. The MUT mice visited the open arm less frequently than the WT mice (WT = 19.33 ± 6.02, *n* = 9; MUT = 12.00 ± 7.33, *n* = 9, *p < 0.05). Error bars represent mean ± SD.

We compared the learning ability of the two groups using the Morris Water Maze test on the 20th week. As shown in Figure 4, the MUT mice took more time to reach the platform over the training test compared to the WT mice from day 1 to day 4, but the difference was not statistically significant (Figure 4B). On day 5, when the previous platform was removed, the MUT mice crossed less frequently to the target quadrant (Figure 4D) and tended to spend less time there than the WT mice, although the difference was not significant (Figure 4C).

We evaluated the anxiety-like behavior between the MUT mice and the WT mice at the age of 20 weeks using the elevated plus maze. Figure 4E shows the traces of WT and MUT mice in the elevated plus-maze in 5 min. The MUT mice spent less time in the open arms (Figure 4F) and also less frequently visited the open arm compared to the WT mice (Figure 4G). Together, the MUT mice had mild memory deficits and anxiety-like behavior at the age of 20 weeks.

### Mutation in the *Timm8a1* gene leads to a reduction in mitochondrial size, correlating with upregulation of mitochondrial fission protein Mtp18

**FIGURE 5:**
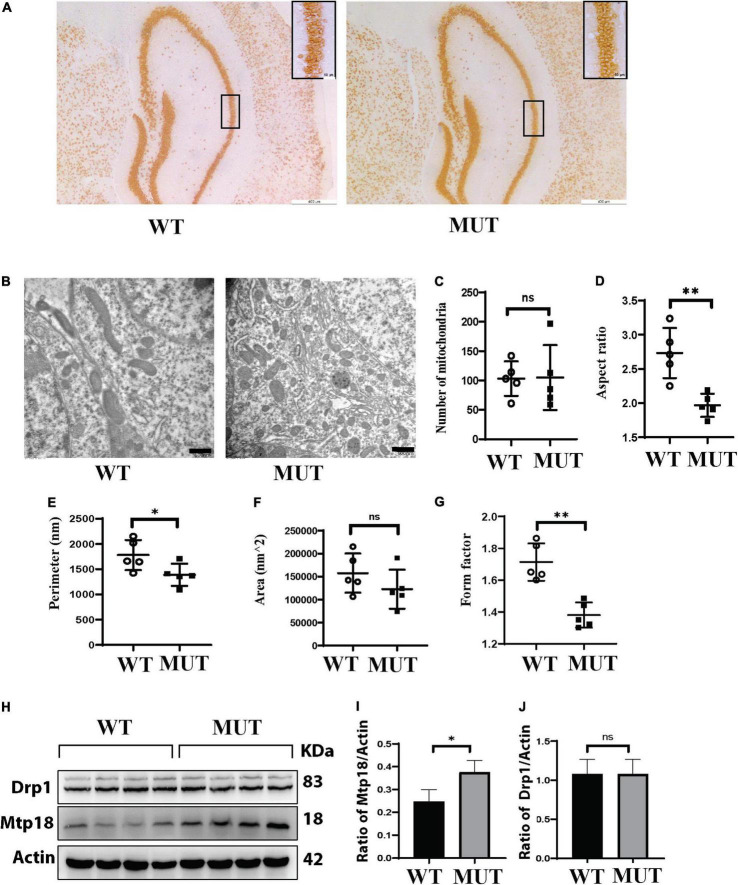
A reduction in the mitochondrial size in the hippocampus of the *Timm8a1*^*I*23*fs*49*X/I*23*fs*49*X*^ MUT mice, correlated to upregulation of mitochondria fission protein Mtp18. **(A)** Histological immunostaining with an anti-NeuN antibody for neural cells in the hippocampus. **(B)** Electron microscopy (EM) images of the hippocampus. **(C)** There was no difference in the mitochondrial numbers between the two genotypes (WT = 103.20 ± 29.38, *n* = 5; MUT = 105.00 ± 55.22, *n* = 5, *p* > 0.05). **(D)** The aspect ratio was significantly decreased in the MUT mice (WT = 2.73 ± 0.36, *n* = 5; MUT = 1.96 ± 0.17, *n* = 5, ^**^p < 0.01). **(E)** The perimeter in the MUT mice was significantly shortened (WT = 1,780.17 ± 299.25, *n* = 5; MUT = 1,387.43 ± 219.27, *n* = 5, *p < 0.05). **(F)** There was a tendency for less area in the MUT mice (WT = 157,451.33 ± 426,57.51, *n* = 5; MUT = 122,523.46 ± 423,66.67, *n* = 5, *p* > 0.05). **(G)** The form factor in the MUT mice was greatly reduced (WT = 1.71 ± 0.11, *n* = 5; MUT = 1.38 ± 0.07, *n* = 5, ^**^p < 0.01). **(H)** Western blotting for the wild-type (WT) and MUT mice at 8 weeks old. **(I)** Mtp18/Actin is upregulated in MUT mice (WT = 0.25 ± 0.05, *n* = 4; MUT = 0.38 ± 0.05, *n* = 4, *p < 0.05). **(J)** There was no difference in dynamin-related protein 1 (Drp1)/Actin between the WT and MUT mice (WT = 1.08 ± 0.19, *n* = 4; MUT = 1.01 ± 0.09, *n* = 4, *p* > 0.05). Error bars represent mean ± SD.

To further investigate the mechanism of memory impairment and anxiety-like phenotypes caused by the Tim8a1 mutation, we first performed a histological analysis of neurons in the hippocampus. As indicated in Figure 5A, there was no significant difference in the number and size of neurons. Second, mitochondrial 2D morphology measurement was taken with the EM images of the hippocampus (Figure 5B). Figure 5C shows there was no difference in the mitochondrial numbers between the two genotypes. However, the mitochondria in the female MUT mice displayed significantly decreased aspect ratio (Figure 5D), shortened perimeter (Figure 5E), a tendency for less area (Figure 5F), and a great reduction in form factor (Figure 5G), compared to those in the WT mice.

Since dynamin-related protein 1 (Drp1) and Mtp18 are important proteins involved in mitochondrial fusion and fission, we assessed levels of these proteins in the hippocampus (Figure 5H). We found that Mtp18 was upregulated in the hippocampus of the MUT mice (Figure 5I), while no obvious change in Drp1 was observed (Figure 5J). Taken together, the DDP1 mutation leads to upregulation of Mtp18, a mitochondrial fission protein, which may cause a reduction in the mitochondrial size and subsequently cause dysfunction of mitochondria in the hippocampus.

### Mutation in the *Timm8a1* gene caused a low fertility rate in female mice

**FIGURE 6:**
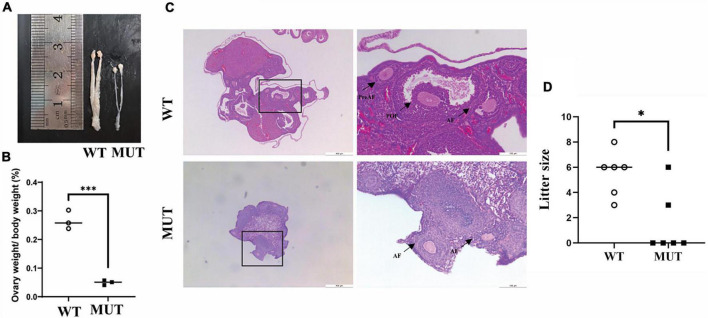
Homologous mutation in the *Timm8a1* gene led to a deficit in female reproductive organs. **(A)** The uterus and ovaries of MUT mice were morphologically smaller than that of the wild-type (WT) control at 24 weeks of age. **(B)** A comparison of the percentage of ovary weight to bodyweight between the WT and MUT mice (WT = 0.27 ± 0.03, *n* = 3; MUT = 0.05 ± 0.01, *n* = 3, ^***^p < 0.001). **(C)** H&E staining of the morphology of the ovary. The preantral follicles (PreAF), antral follicles (AF), and preovulatory follicles (POF) were marked in the figures. **(D)** A comparison of the litter size between the WT and the MUT mice. The Mann–Whitney test was applied to analyze the difference, suggesting a decrease in fertility and litter size in MUT mice compared to WT mice (*n* = 6, *p < 0.05).

Similar to the small sizes of several organs in the male MUT mice (Song et al., 2020), the uterus and ovary organs were remarkably smaller in the MUT mice than in the WT mice (Figures 6A,B). As indicated by the histological analysis with H&E staining in Figure 6C, very few preovulatory follicles were observed in the ovary of the MUT mice. Reproductive experiments showed that the conception rate and litter size were significantly lower in MUT mice than in WT mice (Figure 6D). These results demonstrate the crucial role of Tim8a1 in the development of the reproductive organ in female mice.

## Discussion

Deafness-dystonia-optic neuronopathy syndrome is a rare neurodegenerative disease induced by a mutation in the *TIMM8A* gene encoding the IMS protein DDP1. The main clinical features of DDON syndrome are early onset of sensorineural hearing loss, increased dystonia, ataxia, and psychiatric problems, predominantly in males (Tranebjaerg et al., 1995). A few female patients have late-onset with mild clinical manifestations (Jin et al., 1996; Swerdlow and Wooten, 2001). The pathomechanism is not fully understood.

Previously, we have reported the phenotype of male MUT mice (Song et al., 2020). In this follow-up study, we described in detail the phenotype of female *Timm8a1*^*I*23*fs*49*X/I*23*fs*49*X*^ MUT mice. A comparison between the two sexes of the MUT mice indicates that most phenotypes in the male MUT mice have been recaptured in the female MUT mice, as summarized in Table 1. For example, both the male and female MUT mice were smaller and had lighter weights compared to the WT mice. Similar to the male MUT mice, the female MUT mice showed hearing impairment at 4–5 weeks of age, which mimicked the predominant early-onset hearing loss in male patients with DDON syndrome. The mild coordination problems, memory impairment, and anxiety-like behavior were also observed in the female MUT mice, and the latter two were checked at 20 weeks (Song et al., 2020). Notably, the female MUT mice had a remarkably reduced uterus and ovary. In addition, significantly less mature follicles were found in the female MUT mice, which correlated with a dramatic decline in fertility. Currently, no female patients bearing the homologous mutation of *TIMM8A* have been reported; only a few female carriers present with mild hearing impairment due to X chromosome inactivation. Thus, the additional phenotype in female MUT mice will be difficult to comment on in humans. Compared to hearing impairment, neurological and psychiatric problems are not always present in patients with DDON syndromes (Tranebjaerg et al., 1995). In our follow-up study with three male patients (data not shown), all patients had different degrees of hearing loss since childhood. One patient had severe ataxia, while another patient also had disturbed dystonia.

**TABLE 1:** Comparison of phenotypes between two genders of the Tim8a1 MUT mice.

Tests	*Timm8a1*^*I*23*fs*49*X/Y*^ (Song et al., 2020)	*Timm8a1* ^*I*23*fs*49*X*/*I*23*fs*49*X*^
ABR test	Remarkably damaged (4–5 weeks)	Remarkably damaged (4–5 weeks)
Grip test	No significantly difference (10, 24, and 34 weeks)	No significantly difference (data not shown) (9–10 weeks)
Rotarod test	Significantly difference (10 weeks) No significantly difference (24 and 34 weeks)	Significantly difference (20 weeks)
Balance beam test	No significantly difference in latency (10, 24, and 34 weeks)	No significantly difference in latency (9–10 weeks)
	Significantly increased slips (24 weeks)	Significantly increased slips (9–10 weeks)
Water maze test	Significantly difference (40 weeks)	Significantly difference (20 weeks)
Open field test	Not checked	Significantly difference (20 weeks)
Elevated plus maze	Not checked	No significantly difference (data not shown) (20 weeks)
Reproduction	Not checked	Significantly reductions in the size of reproduction organs and low fertility (10 weeks)

In this study, we elucidated the mechanism underlying the memory impairment and anxiety-like behavior in the mutant mice. The hippocampus is the critical subregion of the brain for memory and emotional function. Although we did not find the general morphological change or loss of neurons in the hippocampus of the mutant female mice, we provided evidence that the mitochondrial size was significantly smaller than that in the WT mice by EM measurement (Figures 5B–G). The mitochondrial dynamic of fusion/fission is implicated in neuronal survival and axon degeneration/regeneration (Youle and van der Bliek, 2012; Morita et al., 2017). Recruiting a cytosolic protein, Drp1, to the mitochondria is the initial step for mitochondrial fission (Wai and Langer, 2016). Mtp18 is thought to coordinate with Drp1 to promote mitochondrial fission. Downregulation of MTFP1 levels causes mitochondrial hyperfusion, while its overexpression reduces mitochondrial size and axon growth suppression (Tondera et al., 2004, 2005). Consistent with this notion, we observed the reduced size of mitochondria in the hippocampus correlated with upregulation of MTFP1, which might lead to mitochondrial dysfunction in the hippocampus and psychiatric problems. Our observation is contrary to other studies in which mutant DDP1 caused increased fusion and elongation of mitochondria in primary fibroblasts from DDON patients (Neighbors et al., 2020). Also, it should be noted that DDP1 binds to Drp1 and promotes mitochondrial redistribution (Arnoult et al., 2005). Here, we did not observe the change in Drp1. Thus, how the loss of Ddp1 leads to the upregulation of MTFP1 remains for further investigation.

There are a few female patients with DDON syndrome, and the reproductive system implication is unclear. The interesting finding here is that the Tim8a1 mutant affects ovarian folliculogenesis in the MUT female mice. Follicles are structures formed by the oocyte and surrounding somatic cells, granulosa, and theca cells. The activated primordial follicles increase through primary, pre-antral, and antral stages. Most follicles at the antral stage endure atretic degeneration, while only a few of them reach the preovulatory stage, waiting for the release of mature oocytes in response to the preovulatory surge of gonadotropins (McGee and Hsueh, 2000; Adhikari and Liu, 2009; Maidarti et al., 2020). Studies in the mouse have shown that mitochondria involved in apoptosis and mitochondrial biogenesis play an important role in follicular atresia (Hsu et al., 1996; Ene et al., 2013; Aiken et al., 2016; May-Panloup et al., 2016). Whether the decrease in the antral follicles in the mouse ovary carrying mutant Tim8a1 was due to apoptosis or dysfunction of mitochondria is still unclear. Nevertheless, the study provides evidence that Ddp1 play a role in the development of follicles in female mice.

In conclusion, the homologous mutation in the *Timm8a1* gene caused the hearing impairment, memory impairment, and anxiety-like behavior, and the latter two phenotypes may be correlated with reduced mitochondrial size regulated by upregulation levels of mitochondrial fission factor, MTFP1. Our ongoing studies focus on follow-up studies with patients having DDON syndrome, the establishment of induced pluripotent stem cells (iPSC) derived from patients, the mechanism of the hearing impairment, and treatment trials in iPSC and animal models.

## Data availability statement

The original contributions presented in this study are included in the article/supplementary material, further inquiries can be directed to the corresponding authors.

## Ethics statement

All animal experiments have been approved by the Animal Ethics Committee of Southern Medical University, China, and conducted following the guidelines for the care and use of Laboratory Animals.

## Author contributions

YFH and SP designed the study. YFH and NO drafted the manuscript. NO and ZC performed the behavior experiments. YiH, YJ, and ZL performed the histological staining analysis. PS and XC performed the ABR test. QL, ZX, KX, and YG participated in the experiment. All authors reviewed the manuscript and approved the submitted version.

## Footnotes

1
https://www.tse-systems.com/
